# Dicoumarol is an effective post-exposure prophylactic for SARS-CoV-2 Omicron infection in human airway epithelium

**DOI:** 10.1038/s41392-023-01511-7

**Published:** 2023-06-10

**Authors:** Yang Peng, Shi-ying Chen, Zhao-ni Wang, Zi-qing Zhou, Jing Sun, Gui-an Zhang, Jia Li, Lei Wang, Jin-cun Zhao, Xiao Xiao Tang, De-Yun Wang, Nan-shan Zhong

**Affiliations:** 1 State Key Laboratory of Respiratory Disease, National Clinical Research Center for Respiratory Disease, Guangzhou Institute of Respiratory Health, The First Affiliated Hospital of Guangzhou Medical University, Guangzhou Medical University, Guangzhou, Guangdong China; 2 Department of Otolaryngology, Infectious Diseases Translational Research Program, Yong Loo Lin School of Medicine, National University of Singapore, Singapore, Singapore; 3 Guangzhou Laboratory, Guangzhou, China

**Keywords:** Drug regulation, Respiratory tract diseases

## Abstract

Repurposing existing drugs to inhibit SARS-CoV-2 infection in airway epithelial cells (AECs) is a quick way to find novel treatments for COVID-19. Computational screening has found dicoumarol (DCM), a natural anticoagulant, to be a potential SARS-CoV-2 inhibitor, but its inhibitory effects and possible working mechanisms remain unknown. Using air-liquid interface culture of primary human AECs, we demonstrated that DCM has potent antiviral activity against the infection of multiple Omicron variants (including BA.1, BQ.1 and XBB.1). Time-of-addition and drug withdrawal assays revealed that early treatment (continuously incubated after viral absorption) of DCM could markedly inhibit Omicron replication in AECs, but DCM did not affect the absorption, exocytosis and spread of viruses or directly eliminate viruses. Mechanistically, we performed single-cell sequencing analysis (a database of 77,969 cells from different airway locations from 10 healthy volunteers) and immunofluorescence staining, and showed that the expression of NAD(P)H quinone oxidoreductase 1 (NQO1), one of the known DCM targets, was predominantly localised in ciliated AECs. We further found that the NQO1 expression level was positively correlated with both the disease severity of COVID-19 patients and virus copy levels in cultured AECs. In addition, DCM treatment downregulated NQO1 expression and disrupted signalling pathways associated with SARS-CoV-2 disease outcomes (e.g., Endocytosis and COVID-19 signalling pathways) in cultured AECs. Collectively, we demonstrated that DCM is an effective post-exposure prophylactic for SARS-CoV-2 infection in the human AECs, and these findings could help physicians formulate novel treatment strategies for COVID-19.

## Introduction

The COVID-19 pandemic has frequently produced highly transmissible variants of severe acute respiratory syndrome coronavirus 2 (SARS-CoV-2), such as the Omicron identified in South Africa in November 2021.^1^ The Omicron variant is characterised by its high transmissibility and a short incubation period, which allows it to spread faster than the previous variants (e.g., Alpha, Beta, Gamma and Delta) even in populations with high vaccination rates.^2^ Thus, scientists worldwide are researching drugs to inhibit SARS-CoV-2. Current therapeutic agents for treating SARS-CoV-2 infection include Pfizer’s oral drug combination nirmatrelvir/ritonavir (Paxlovid), repurposed small molecules such as remdesivir and virus-specific monoclonal antibodies.^3,4^ However, high cost, limited availability, and logistical challenges have limited the public health impact of these treatments.^5^ Repurposing pre-existing drugs is a quick way to find effective therapies for the Omicron variants during a pandemic, as their dosage, safety, and mechanism of action have already been well established.^6^

Dicoumarol (DCM), derived from natural origin, is considered a safe human oral anticoagulant prescribed in clinics for decades.^7^ Studies have already shown that DCM exhibits wide-ranging antiviral activities against Hepatitis B virus (HBV) and human immunodeficiency virus (HIV).^8,9^ One mode of action of DCM is to inhibit the NAD(P)H: quinone oxidoreductase 1 (NQO1), an intracellular quinone oxidoreductase that reduces reactive oxygen species production, by competing with NADH for the binding site.^10,11^ It has been reported that NQO1 acts as a key mediator of neutrophil elastase-regulated oxidant stress.^12^ Besides, DCM has been used as a natural anticoagulant due to its ability to antagonise the blood clotting process by inhibiting the vitamin K epoxide reductase complex subunit 1 (VKORC1).^10^ VKORC1 encodes the vitamin K epoxide reductase enzyme necessary for vitamin K biosynthesis.^13^ Dofferhoff et al. found reduced vitamin K levels in COVID-19 patients associated with poor prognosis.^14^ Recently, DCM has been identified in several virtual screening studies as a potential drug for the treatment of COVID-19.^15–17^ However, DCM’s inhibitory effects and possible working mechanisms in SARS-CoV-2 (especially in Omicron variants) infection remain unknown.

The airway epithelium is the first line of airway defense against pathogen infection. This is achieved through mucociliary clearance and production of several defense proteins such as mucins, cytokines, and chemokines.^18^ Dysfunction of mucociliary clearance can promote mucus accumulation in the respiratory tracts, perpetuating tissue damage and leading to disease development (e.g., chronic rhinosinusitis and asthma).^19,20^ To our knowledge, SARS-CoV-2 targets the ciliated and club cells via the binding of the spike protein to the angiotensin-converting enzyme 2 (ACE2) receptor in the airway epithelium.^21,22^ The massive replication of SARS-CoV-2 compromises mucociliary clearance, thereby facilitating its spread to the lungs and paving the way for secondary infections.^23^ Omicron variants have been reported to dramatically accelerate the spread via the ciliary transport/microvilli reprogramming pathway in airway epithelium, which explains the increase in their attack rate compared to previous variants, including the wild-type virus and Delta variants.^23^ Decreasing the viral load by blocking viral replication could prevent disease progression and limit the infectivity of COVID-19 patients.^24^ Therefore, developing new antiviral strategies, including drugs preventing viral entry and/or replication in the Omicron-infected airway epithelial cells (AECs), is important.

In this study, we sought to determine whether DCM can regulate the anti-Omicron function of primary human AECs cultured at an air-liquid interface (ALI) condition. Using the time-of-addition and drug withdrawal assays, we demonstrated that DCM, added shortly after an infection, suppresses SARS-CoV-2 replication in primary AECs. In addition, we also assessed the relationship between the expression of the DCM targets (NQO1 and VKORC1) and SARS-CoV-2 infection in AECs by using single-cell sequencing analysis, and defined the underlying mechanism of DCM in anti-Omicron replication by using transcriptome sequencing. Our findings support the development of DCM as a post-exposure therapeutic for SARS-CoV-2 infection, which could help physicians formulate a novel strategy for COVID-19 patients by repurposing existing drugs.

## Results

### The characteristics of Omicron infection in AECs and the inhibitory effect of DCM

**Fig. 1:**
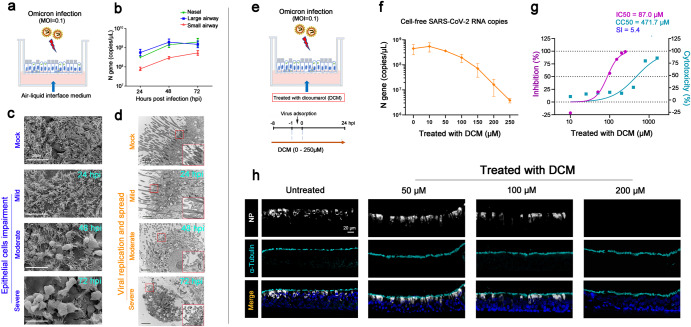
The characteristics of Omicron infection in airway epithelial cells (AECs) and the inhibitory effect of dicoumarol (DCM) on viral infection. Cultured primary AECs were infected with SARS-CoV-2 Omicron BA.1 at a multiplicity of infection (MOI) of 0.1 (**a**). The cell-free SARS-CoV-2 N gene were measured in the supernatant of cultured AECs derived from nasal (*n* = 4), large airway (*n* = 5) and small airway (*n* = 4) (**b**). Representative images of AECs with Omicron infection were observed by scanning electron microscopy (**c**) and transmission electron microscopy (**d**). The basolateral side of the AECs culture was treated with DCM at concentration of 0 μM to 250 μM (*n* = 3–6 at each concentration) (**e**). The antiviral effect (**f**, **g**) and cell cytotoxicity (**g**) of DCM treatment in AECs were measured. Representative immunofluorescence images showed that DCM markedly reduced the proportion of infected cells (N protein positive) at concentration of 200 μM (**h**). Data are represented as mean ± SEM

We cultured primary AECs derived from human nasal (*n* = 4), large airway (*n* = 5), and small airway (*n* = 4) at ALI conditions, and then infected them with Omicron BA.1 at a multiplicity of infection (MOI) of 0.1 (Fig. 1a). Examination of the cell-free SARS-CoV-2 N gene in the supernatant found increased viral gene copies in both the 48 h and 72 h post-infection cultures, as compared with that in the 24 h post-infection culture (Fig. 1b). In addition, we found higher viral N gene copies in AECs from the nasal and large airway, as compared with that from the small airway (Fig. 1b and Supplementary Fig. 1). Representative scanning electron microscopy (SEM) images showed that viral infection resulted in moderate to severe impairment and shedding of infected AECs into the airway surface microenvironment at 48 h and 72 h post-infection (Fig. 1c). Transmission electron microscopy (TEM) revealed that the vesicles in infected cells were filled with viral particles of various sizes and spherical or multiform morphology (Fig. 1d). Most viral particles were in the cytoplasm and fewer viral particles were on microvillar structures at 24 h post-infection, then a large number of newly generated virus particles accumulated outside the cell membrane at 48 h and 72 h post-infection (Fig. 1d). These observations of SEM and TEM suggested that most of the obvious viral exocytosis and viral spread occurred after 24 h post-infection.

To investigate the effect of DCM on AECs with Omicron infection, the basolateral side of the ALI culture was pre-treated with a series of concentrations of DCM (0–250 μM) for 8 h before viral infection, and DCM was maintained in the medium until 24 h following infection (Fig. 1e). Vehicle control was treated with NaOH, the dissolving medium for DCM, at concentrations of 0–1 mM (Supplementary Fig. 2). We did not note marked differences in pH value (alteration of pH value < 0.3) when the culture medium was treated with DCM (at 50–400 μM) or NaOH (at 0.5–4.0 mM) (Supplementary Table 1). By using qRT-PCR, we noted that DCM potently reduced the levels of cell-free SARS-CoV-2 N gene copies in a dose-dependent manner with the half-maximal inhibitory concentration (IC50) value of 87.0 μM (Fig. 1f, g). The 50% cytotoxic concentration (CC50) values of DCM were computed to be 471.7 μM in AECs (Fig. 1g), and the selectivity index (SI = CC50/IC50) of DCM was 5.4. Representative immunofluorescence (IF) images showed that DCM at concentrations of 200 μM markedly attenuated a proportion of AECs with SARS-CoV-2 infection (N protein positive cells) (Fig. 1h). We further revealed that treatment of DCM with a concentration of 200 μM could also inhibit the infection of multiple SARS-CoV-2 variants (including Alpha, Delta and Omicron BQ.1/XBB.1) in cultured AECs at 24 or 48 h post-infection (Supplementary Fig. 3).

Overall, these results indicate that DCM has potent antiviral activity against SARS-CoV-2 infection in AECs.

### Early treatment of DCM markedly inhibited the Omicron replication in AECs

A recent paper^23^ published in the journal *Cell* has shown in primary nasal epithelial cell cultures the course taken by of SARS-CoV-2 to penetrate the airway epithelial barrier, which includes: 1) viral adsorption and entry via ACE2 receptor of ciliated AECs; 2) viral replication in infected cellular cytoplasm; 3) viral exocytosis from the microvilli back into the mucus layer at 24 h post-infection; and 4) viral spread via mucociliary transport.

**Fig. 2:**
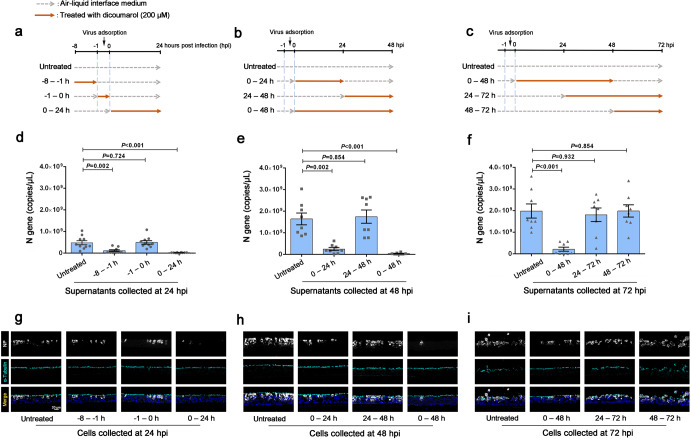
Dicoumarol (DCM) early treatment markedly inhibited the Omicron replication in cultured airway epithelial cells (AECs). Experimental procedures of time-of-addition assay and withdrawal assay for discriminating the anti-Omicron stage of 200 μM DCM in cultured AECs at indicated time points (*n* = 4–5 per group, tested in duplicate) (**a**–**c**). The cell-free SARS-CoV-2 N gene expression in supernatants of cultured AECs were measured by quantitative polymerase chain reaction (**d**–**f**). The SARS-CoV-2 N protein expression in infected cells were detected by immunofluorescence staining (**g**–**i**). Data are represented as mean ± SEM

To investigate the antiviral stage of DCM, we performed a time-of-addition assay and a drug withdrawal assay. The primary AECs were treated with 200 μM DCM and infected with Omicron BA.1, at 24 h (Fig. 2a), 48 h (Fig. 2b), and 72 h (Fig. 2c) respectively. As shown in Fig. 2d, g (in 24 h post-infection assay), we found that: 1) no inhibition was observed when DCM was solely added during the 1 h of viral adsorption and entry (−1 to 0 h); 2) both the viral N genes and N protein were suppressed by DCM when the compound was incubated before (−8 to −1 h) or after virus adsorption (0–24 h). These results suggest that viral replication has been inhibited by early treatment of DCM.

**Fig. 3:**
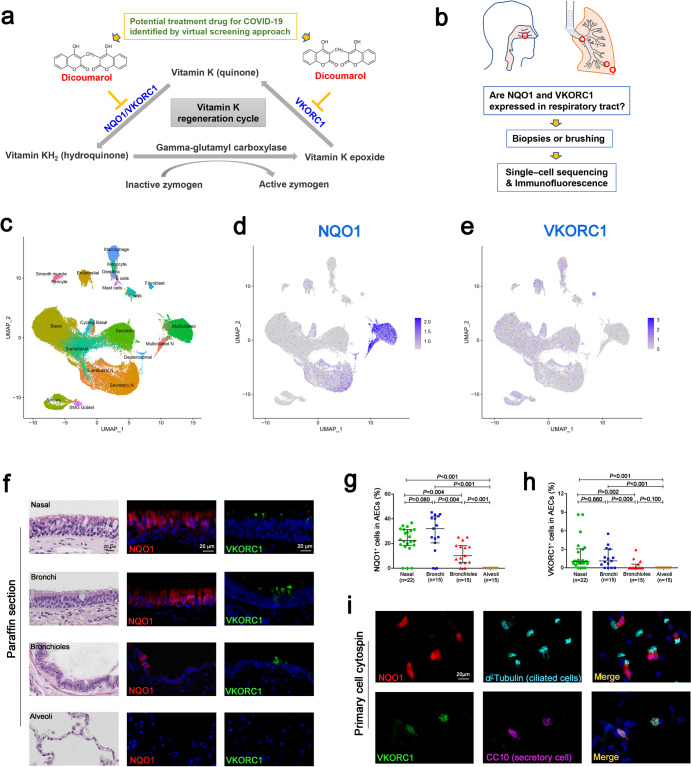
Dicoumarol (DCM)’s targets were expressed in ciliated cells and secretory cells within airways. The chemical structure of DCM and the roles of DCM’s recognized targets (NQO1 and VKORC1) in the recycling of vitamin K (**a**). Airway samples from distinct regions were collected (**b**) for detecting the RNA and protein expression patterns of NQO1 and VKORC1 by using single-cell sequencing analysis (**c**–**e**) and immunofluorescence staining (**f**–**i**), respectively. Data are represented as median (1st, 3rd quartile)

We have revealed that viral exocytosis and viral spread usually occur after 24 h post-infection, as mentioned above (Fig. 1b-d). As shown in Fig. 2e, f (in 48 or 72 h post-infection assay), we did not find distinct suppression of virus infection with delayed incubation with the following: (1) DCM added between 24 and 48 h, with samples collected at 48 h (Fig. 2e, h); (2) DCM added between 24 and 72 h, with samples collected at 72 h (Fig. 2f, i); (3) DCM added between 48 and 72 h, with samples collected at 72 h (Fig. 2f, i). These results indicate that DCM treatment is unlikely to inhibit viral exocytosis and viral spread. Furthermore, as shown in Fig. 2e, f (in 48 or 72 h post-infection assay), we noted recovery of viral replication detected by qRT-PCR and IF staining when: (1) the compound was incubated between 0 and 24 h but removed between 24 and 48 h, and samples were collected at 48 h (Fig. 2e, h); (2) the compound was incubated between 0 and 48 h but removed between 48 and 72 h, and samples were collected at 72 h (Fig. 2f, i). These results suggest that DCM treatment is unlikely to directly eliminate the viral particle.

In summary, we demonstrated that the early treatment with DCM can markedly inhibit Omicron replication in AECs, but it is unlikely that DCM can affect viral absorption, viral exocytosis, viral spread, or directly eliminate viruses from the infected cells.

### DCM’s targets were expressed in ciliated cells and secretory cells within airways

Next, we explored the potential molecular mechanism for the inhibitory effects of DCM on AECs with Omicron infection. DCM has been recognised as an inhibitor of two targets, NQO1 and VKORC1, in the recycling of vitamin K metabolism (Fig. 3a).^10^ However, the cell-specific expression patterns of NQO1 and VKORC1 in AECs remain unclear. Here, we examined the expression and distribution of NQO1 and VKORC1 within the AECs in samples from distinct airway regions (nasal, bronchi, bronchioles and alveoli) by using single-cell sequencing analysis and IF staining (Fig. 3b).

Based on the published database,^25^ the cell types were assigned to various clusters using well-established sets of marker genes (Fig. 3c and Supplementary Data 1). The basal, secretory and ciliated AECs collectively represented most of the total surface AECs. The ciliated AECs comprising a subcluster of cells, which could only be detected in nasal samples, were labelled as “Multiciliated N” (Fig. 3c). As shown on the feature plot, the expression of NQO1 was predominantly in ciliated AECs (Fig. 3d), and restricted expression of VKORC1 was detected in most AECs (Fig. 3e).

The distribution of NQO1 and VKORC1 proteins within the respiratory tracts was further explored and analyzed by using IF staining (Fig. 3f). The median (1^st^, 3^rd^ quartile) percentages of NQO1 positive cells in AECs were 22.49% (18.45%, 31.34%), 32.10% (20.59%, 42.11%), 10.17% (4.55%, 18.39%), and 0% (0%, 0%) in epithelial areas of the nasal, bronchi, bronchioles and alveoli, respectively (Fig. 3g). In addition, we noted a rare presence of VKORC1 positive cells in the AECs, with median (1^st^, 3^rd^ quartile) percentages of 1.05% (0.62%, 3.08%), 1.12% (0%, 3.03%), 0% (0%, 0.61%) and 0% (0%, 0%) in the epithelial areas of the same four respiratory tracts regions, respectively (Fig. 3h). The percentages of both NQO1^+^ AECs and VKORC1^+^ AECs were significantly lower in the bronchioles, compared with that in the nasal and bronchi (*P* < 0.05), but their presence in the nasal and bronchi was comparable (*P* > 0.05) (Fig. 3g, h).

We then examined the co-localisation of NQO1 and VKORC1 as well as the AECs markers (CC10 in secretory cells, α-tubulin in ciliated cells and KRT5 in basal cells) on cytospin slides of cells obtained through nasal and bronchial cytology brushing. We thereby confirmed that NQO1 was predominantly expressed in partially ciliated AECs, while VKORC1 was predominantly expressed in partial secretory AECs (Fig. 3i and Supplementary Fig. 4).

### The relationship among NQO1/VKORC1 expression, COVID-19 severity and SARS-CoV-2 infection in cultured AECs

**Fig. 4:**
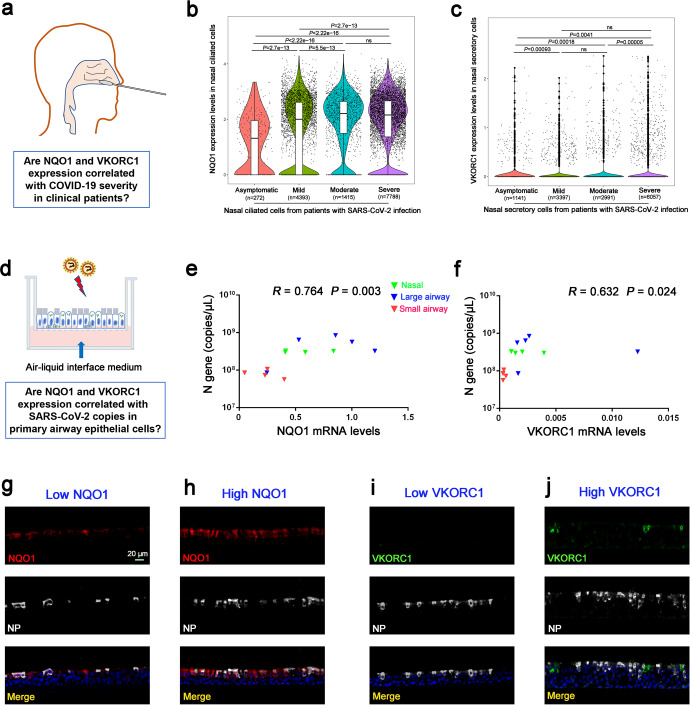
The relationship among the NQO1/VKORC1 expression, COVID-19 severity in patients and SARS-CoV-2 infection in cultured airway epithelial cells (AECs). The expression levels of NQO1 and VKORC1 in nasal brushing cells from COVID-19 patients were analyzed by single-cell RNA sequencing dataset (**a**–**c**). The correlation between NQO1/VKORC1 expression and SARS-CoV-2 N gene/protein expression in cultured AECs was analyzed by quantitative polymerase chain reaction (**d**–**f**) and immunofluorescence staining (**g**–**j**)

Having revealed the expression profile of DCM’s targets (NQO1 and VKORC1) in AECs, we then explored whether the NQO1 and VKORC1 expressions in AECs correlated with the COVID-19 severity in clinical patients (Fig. 4a). Our analysis of a publicly available RNA-seq database (https://covid19cellatlas.org) found a significant positive relationship between the NQO1 expression in nasal ciliated AECs and disease severity in patients with SARS-CoV-2 infection (asymptomatic subjects< mild patients< moderate/severe patients, all *P* < 0.05) (Fig. 4b). In addition, we found that VKORC1 expression in nasal secretory AECs was higher in symptomatic patients than in asymptomatic patients (all *P* < 0.05), but was comparable among patients with mild, moderate and severe COVID-19 symptom (all *P* > 0.05) (Fig. 4c).

Moreover, we explored whether the NQO1 or VKORC1 expressions correlated with the cell-free SARS-CoV-2 N gene copies in our Omicron-infected AEC cultures (Fig. 4d). We found that the viral gene copies had a significant positive correlation with both NQO1 expression levels (*R* = 0.764, *P* = 0.003) (Fig. 4e) and VKORC1 expression levels (*R* = 0.632, *P* = 0.024) (Fig. 4f). IF staining was further performed to explore whether the NQO1 or VKORC1 expressions correlated with the SARS-CoV-2 N protein expression in infected AECs. We noted that less amount of N protein was observed in AECs with low NQO1 expression (Fig. 4g) than in those with high NQO1 expression (Fig. 4h), and similar pattern was found for VKORC1 expression (Fig. 4i, j).

These findings suggest that inhibition of NQO1 and VKORC1 expressions in AECs may be a useful treatment to reduce the severity of the COVID-19.

### Potential target and signalling pathways of DCM against Omicron in AECs

**Fig. 5:**
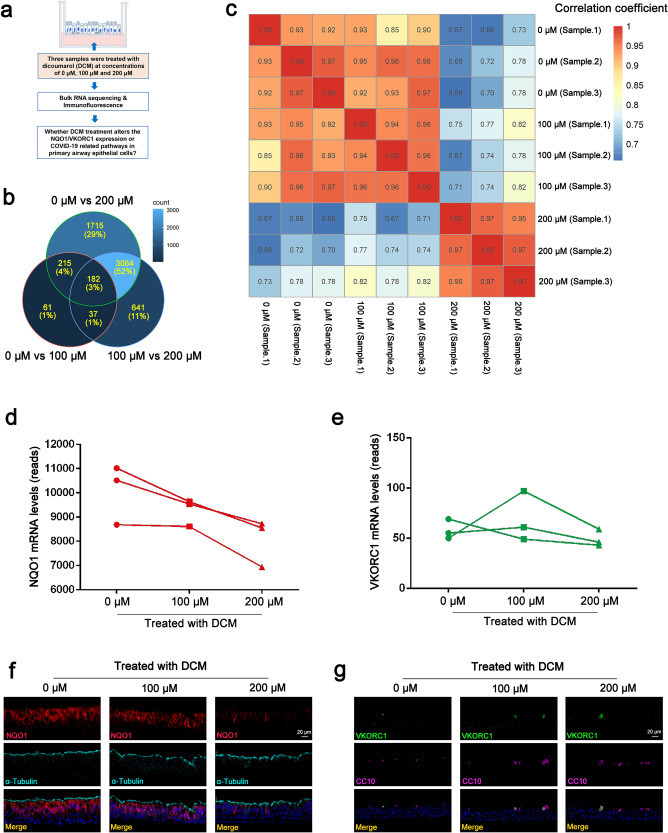
Dicoumarol (DCM) treatment alters gene expression of cultured airway epithelial cells (AECs). Transcriptome sequencing was performed to detect the gene expression pattern in AECs treated with DCM at concentrations of 0, 100 and 200 μM (**a**). Venn diagram representing the overlaps of differentially expressed genes among the three pairwise comparisons (**b**). R package corrplot suggested there was strong differential gene expression in AECs treated with 200 μM DCM compared with those treated with 0 μM and 100 μM DCM (**c)**. The mRNA expression levels of NQO1 (**d**), but not that of VKORC1 (**e**), were decreased with DCM treatment, in a dose-dependent manner. Representative immunofluorescence images showed the protein expressions of NQO1 (**f**) and VKORC1 (**g**) in AECs treated with DCM

We then investigated the effects of DCM treatment (at a concentration of 0, 100 and 200 μM) on three cultured AEC samples at the molecular level by using transcriptome sequencing and attempted to understand the potential anti-Omicron mechanism of DCM (Fig. 5a). Venn diagram shows the overlap between the differentially expressed genes among the three pairwise comparisons (Fig. 5b). To easily assess co-expression and anti-correlation between genes, we also visualised the co-expression potential with the R package corrplot (Fig. 5c). These results showed a strong differential gene expression upon treatment with 200 μM DCM compared with treatment with 0 μM or 100 μM DCM, whereas the gene expression at concentrations of 0 μM and 100 μM were comparable. In addition, transcriptome sequencing and IF staining further revealed decreased expression of NQO1, but not of VKORC1, in a dose-dependent manner with DCM treatment (Fig. 5d–g**)**. These data suggest the anti-Omicron activity of DCM in AECs is likely related to the suppression of NQO1 expression.

**Fig. 6:**
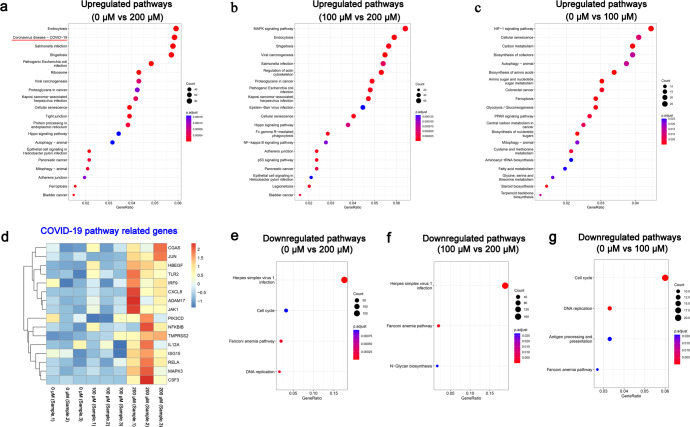
Potential signalling pathways of Dicoumarol (DCM) against Omicron in cultured airway epithelial cells (AECs). Among AECs treated with DCM at concentrations of 0 μM, 100 μM and 200 μM, the Kyoto Encyclopedia of Genes and Genomes (KEGG) pathway analysis was performed to identify the upregulated pathways (**a**–**c)** and downregulated pathways (**e**–**g**) between groups, and the heatmap showed the representative genes expression related to COVID-19 signalling pathway (**d**)

Next, Kyoto Encyclopedia of Genes and Genomes (KEGG) analysis revealed that signalling pathways associated with SARS-CoV-2 disease outcomes (e.g., endocytosis and COVID-19 signalling pathways) were significantly upregulated after treatment with 200 μM (vs. 0 μM) DCM (Fig. 6a), but not after treatment with 100 μM (vs. 0 μM) DCM (Fig. 6b, c). Heatmap showed upregulation of genes (e.g., *TMPRSS2*, *JUN* and *CXCL8*) related to the COVID-19 signalling pathway upon DCM treatment at a concentration of 200 μM, compared with concentrations of 0 μM and 100 μM (Fig. 6d). In addition, KEGG analysis revealed downregulation of a signalling pathway associated with virus infection (herpes simplex virus 1 infection) after treatment with 200 μM (vs. 0 μM or 100 μM) DCM **(**Fig. 6e–g). Taken together, our data provided preliminary evidence that DCM at a therapeutic dose of 200 μM can alter the progression of SARS-CoV-2 infection in AECs.

## Discussion

Respiratory symptoms are prominent complaints in most SARS-CoV-2 infections, and progressive respiratory dysfunction is a major feature of fatal COVID-19. The presence of the Omicron variant has led to a more significant escape from immune protection elicited by previous SARS-CoV-2 infection.^26^ So far, there is limited effective treatment for patients who fall ill despite vaccination or other preventive measures. In this study, we demonstrated that DCM, a natural anticoagulant, inhibits SARS-CoV-2 Omicron variant infection at early phases after viral absorption in AECs. Moreover, we demonstrated that it is unlikely that DCM can affect viral absorption, viral exocytosis, viral spread, or directly eliminate viruses from the infected cells. We further explored the mechanism underlying action of DCM in anti-Omicron replication. NQO1 expression levels showed a significant positive correlation with the severity of COVID-19 in patients and virus copies in cultured AECs; Also, DCM treatment can attenuate NQO1 expression. Therefore, our data support further testing and development of DCM as a post-exposure prophylactic or early-phase therapeutic strategy for SARS-CoV-2 infection.

In response to the severe global pandemic, scientists worldwide have unleashed an unprecedented response to finding effective treatment strategies for COVID-19.^27^ There are several antiviral treatment options that can block the SARS-CoV-2 viral replication and thereby prevent COVID-19 disease progression. The oral drug combination nirmatrelvir/ritonavir (Paxlovid) was strongly recommended as a therapeutic option for patients with non-severe COVID-19 who are at the highest risk of hospitalisation.^28^ The active substance nirmatrelvir blocks the activity of an enzyme needed by the virus to multiply. Co-administering nirmatrelvir with low-dose ritonavir slows the breakdown of nirmatrelvir, thereby increasing its therapeutic benefit.^3^ Paxlovid must be started within five days of symptoms onset and taken for a duration of five days. However, access to nirmatrelvir is limited in low-income countries, and ritonavir has multiple drug-drug interactions warranting specialised assessment before prescription.^29,30^ Remdesivir targets the RNA-dependent RNA polymerase, an essential enzyme for viral RNA replication and a promising drug target for COVID-19.^31^ Early combined treatment using remdesivir plus dexamethasone in COVID-19 patients requiring supplemental oxygen therapy induces a rapid SARS-CoV-2 clearance, reduces the risk of 30-day mortality and prevents clinical progression of the disease.^32^ However, remdesivir must be administered intravenously, limiting its widespread use during the pandemic.^33^ Recently, an oral analogue of remdesivir, VV116, has been developed to address this issue. Early administration of oral VV116 was non-inferior to Paxlovid in shortening the time to sustained clinical recovery in participants who were at high risk for progression to severe COVID-19 disease.^33^ In addition, VV116 also had fewer safety concerns than Paxlovid. However, developing these drugs could take years, and faster approaches must be taken to combat this devastating pandemic. Repurposing existing human drugs is a quick solution to this developing demand, as their dosage, safety, and mechanism of action have already been well established.

Cell-based or computational screening techniques have been successfully utilized to screen existing drugs for repurposing against COVID-19.^34^ However, most drug repurposing is still at the computational level, lacking experimental validation and requiring investigational clinical trials. DCM is considered a safe compound for human oral consumption and represents an important advantage as compared with new synthetic drugs. Moreover, its production is less complicated and less costly because fewer downstream operations would be needed. DCM is considered as the “parent” of the widely used anticoagulant drug, warfarin.^7^ For preventing or treating harmful blood clots in adults, the common dose of DCM is 25 to 200 mg per day, adjusted according to blood tests. Clinically, the target international standardised ratio is 2.0 to 3.0, which can not only ensure the DCM treatment effect but also maintain the risk of haemorrhage at a low level. In clinical practice, several large cohort studies had reported that anticoagulation treatment of patients using warfarin before COVID-19-related hospitalization improved therapeutic outcomes and reduced admission to intensive care unit.^35–38^ Surprisingly, it has also been observed that warfarin users were less likely to be testing positive for SARS-CoV-2 infection.^39^ However, in patients not under anticoagulation therapy before hospitalisation, anticoagulation started during hospitalisation was not associated with a better prognosis.^35^ Consistent with the results from these clinical studies, our in vitro findings provided preliminary evidence and indicated that early treatment with DCM can markedly inhibit SARS-CoV-2 replication in AECs. Since DCM is an anticoagulant and a high dose of it can result in uncontrolled bleeding, the DCM dose to use for effective inhibition of Omicron replication in airway epithelium should be carefully evaluated clinically.

In addition to its anticoagulant properties, DCM and its derivatives have also gained much attention due to their other bioactive properties, including anticancer, antimicrobial, and antiviral activities.^7,40^ However, the mechanisms of action underlying them are mostly unclear, and additional research is also needed to unravel them. Cheng and colleagues have provided evidence that NQO1 has a role in HBV replication through regulating the stability of HBV X protein; DCM treatment and NQO1 knockdown significantly inhibited the transcriptional activity of the intracellular HBV replication intermediate termed covalently closed circular DNA.^8^ Lata et al. reported that NQO1 stabilizes transactivation protein (Tat), the single most important protein of HIV-1, for enhancing replication of the virus, in a dose-dependent manner; DCM treatment potently induced degradation of the Tat protein and inhibited HIV-1 replication.^9^ These data have implications for understanding the regulatory mechanism of DCM in viral infective diseases. NQO1 plays an irreplaceable function in the process of material and energy metabolism in cells, including superoxide elimination, p53 modulation, proteasomal degradation, and detoxification of xenobiotics, all of which could be inhibited by DCM.^41^ In our study, we have mapped the transcriptional profile of DCM-treated AECs from uninfected individuals with no known airway disease or smoking history. One interesting and novel observation from our study was the enrichment of the “COVID-19 signalling pathway” by DCM treatment at 200 μM but not at 100 μM, which was characterised by altered expression of several associated genes, including *TMPRSS2*, *ADAM17*, and *TLR2*. The differences in transcriptome levels brought about by varying concentrations of DCM (200 μM and 100 μM) suggest the importance of an adequate dose of DCM for effective intervention of the Omicron virus infection. DCM also modulates several anti-infection signalling pathways (Viral carcinogenesis, Salmonella infection, and Shigellosis), implicating that it may interrupt the process of multiple viruses invasion and has broad anti-infection activity against different pathogens. Furthermore, the cellular senescence pathway is characterised as a state of stable growth arrest and resistance to apoptosis, and may play an important role in attenuating the AECs damage and immune pathogenesis associated with SARS-CoV-2.^42^ Our study results suggested that DCM might influence AECs by altering these signalling pathways. This has inspired ongoing studies to elucidate further the anti-Omicron mechanism of DCM in both animal models and in human clinical trials.

In our study, the reconstituted ALI-cultured primary AECs not only allow visualisation of SARS-CoV-2’s high levels of replication and destructive nature but also provide a model for assessing the antiviral activity of DCM. The initial steps of SARS-CoV-2 infection involve the specific binding of the virus spike protein to the cellular entry receptor. During the intracellular life cycle, viruses express and replicate their genomic RNA to produce full-length copies that are incorporated into newly produced viral particles.^43^ Then viruses induced the highly extended microvilli to penetrate the periciliary layer, and concatenated chains of virus formed on the extended microvillar structure.^23^ These viral chains appear to accumulate in the mucus layer and infect other cells via mucociliary transport. In the current study, time-of-addition and withdrawal assays allow us to better understand DCM’s effect on the infectious phase. Our results showed DCM did not affect on viral absorption, viral exocytosis, viral spread, or directly eliminate viruses from the infected AECs. However, in vivo studies will be needed to reveal additional insights into the DCM’s inhibition of Omicron’s replication in AECs.

There are limitations to our study. First, it is evident that physical barriers, airway secretions, and host defense mechanisms of pseudostratified AECs significantly hinder the delivery and efficacy of the siRNA reagents.^44^ This creates an impediment in studies of the AECs, and cell type-specific transgenic systems are not yet available for AECs populations, especially in ciliated AECs.^45^ Therefore, we did not address the role and regulatory mechanism of NQO1 and VOKRC1 in SARS-CoV-2 replication through such means as gain and loss of functional experiments. Second, our model lacks resident microbial flora and inflammatory cells (dendritic cells, macrophages, lymphocytes, etc.) ordinarily present in the airway mucosa, which may interact with the airway epithelium to modulate the local immune response against virus infections. Furthermore, our study was conducted in vitro, and further research is necessary to validate these findings in animal models and, eventually, in human clinical trials. Despite the limitations of the present study, we are embarking on serial studies, and hopefully, our ongoing efforts will further address the unmet medical needs.

In conclusion, our study shows that DCM treatment is effective in controlling Omicron replication in AECs at the early stage of infection. These findings could help physicians formulate novel treatment strategies for patients with COVID-19.

## Materials and methods

### Subject recruitment and sample processing

Approval of the study protocol was obtained from the Institutional Review Board of the First Affiliated Hospital of Guangzhou Medical University (Reference number: 2018-92). All study participants provided informed consent. In order to perform the Omicron infection in primary AECs and to detect the NQO1 and VKORC1 distribution in human paraffin samples, we obtained biopsies including samplings from the inferior turbinate (*n* = 25) of patients undergoing septoplasty due to anatomic variation, bronchi (*n* = 19) and peripheral lungs (*n* = 20) from healthy controls who underwent lung transplantation or from patients with solitary peripheral carcinoma who underwent lung resection, respectively. All recruited subjects have no history of smoking, chronic rhinosinusitis, asthma, or chronic obstructive pulmonary disease.

### IF staining

Protein expression was examined by using IF staining on the slides prepared from paraffin sections, the membranes of transwell inserts, and primary single-cell cytospin. Slides were subject to heat-induced antigen retrieval in Tris-EDTA buffer (pH 9.0) at 95 °C for 15 min in a microwave oven and cooled at room temperature. The slides were then incubated with primary antibodies of NQO1, VKORC1, SARS-CoV-2 Nucleoprotein, α-tubulin for ciliated cells, club cell 10 kDa protein (CC10) for club cells, keratin 5 (KRT5) for basal cell, respectively. Next, the slides were incubated with Alexa Fluor 488-, 555-, and 647-conjugated secondary antibodies at 37 °C for 1 h at 1:500 dilution. Finally, the nuclei were stained with 4’-6-diamidino-2-phenylindole (Life Technologies, Carlsbad, CA, United States). Images were acquired with fluorescence microscopy (Leica DM6, Wetzlar, Germany). Details of antibodies used are shown in Supplementary Table 2.

The total number of AECs was assessed by manually counting all cellular nuclei located in five epithelial areas. The percentage of NQO1^+^ AECs and VKORC1^+^ AECs was calculated as the number of positively stained cells divided by at least 200 AECs at high power fields and then multiplied by 100% in a blinded manner.

### Cytospin preparation

AECs from nasal brushing were fixed in 4% formaldehyde at room temperature for 10 min, followed by two rounds of washing with Dulbecco’s phosphate-buffered saline, and centrifuged. Next, cytospin preparations were prepared at 500 rpm for 5 min with mild acceleration by using a Shandon Cytospin 4 Cytocentrifuge (Thermo Scientific; Thermo Fischer Scientific, Waltham, MA).

### Air-liquid interface culture system

Primary human AECs were isolated from freshly resected inferior turbinate (*n* = 4), bronchi (large airway, *n* = 5) and peripheral lung (small airway, *n* = 4). The epithelial progenitor cells were cultured in the cell culture system, and the cell medium was changed every 3 days. The cells were cultured at ALI and infected with SARS-CoV-2 at the fully differentiated time point (3–4 weeks).^22,23^

### SARS-CoV-2 infection and DCM treatment

The ALI-cultured cells on the apical chamber of the transwell inserts were infected with the SARS-CoV-2 variants [including Alpha, Delta and Omicron (BA.1, BQ.1 and XBB.1)] viral inoculum at an MOI of 0.1. The inoculated plates were incubated for 1 h at 37 °C with 5% CO_2_. At the end of the incubation, the inoculum was removed from the apical chamber. The basolateral side of the AECs culture was pre-treated with DCM at concentrations of 0–250 μM (dissolved in 0–1 mM NaOH) for 8 h prior to viral infection, and DCM was maintained in the media until 24 h post-infection.

The viral RNA was extracted from the cell supernatant (derived from apical of ALI culture) by using the EZ-press Viral RNA Purification Kit (EZB-VRN1, EZBioscience, Roseville, MN). SARS-CoV-2 N gene copy number was determined using a novel coronavirus (2019-nCoV) nucleic acid diagnostic kit (PCR-Fluorescence Probing) (Daan Gene, Guangzhou, China) according to the manufacturer’s protocol. The percentage of inhibition of SARS-CoV-2 N gene copies in supernatant of cultured AECs was estimated for each drug concentration, and the IC50 was determined by using Graphpad Prism (version 8.0).^46,47^

In addition, the infected cells were harvested to assess N protein expression using SARS-CoV-2 antibodies via IF staining.

### Cell viability assay

In order to determine the cellular viability following DCM treatment, Cell Counting Kit-8 (CCK-8) (purchased from Beyotime Biotechnology, China) was used for the cultured AECs. The cytotoxicity detection assay was performed according to manufacturer’s instructions with minor adjustments.^48^ In brief, after exposure to 24 h DCM treatment at concentrations ranging from 0 to 1600 μM, 110 μL of WST-8 solution (10 μL CCK-8 solution plus 100 μL of ALI differentiation medium) was added to the surface of the cell layer in transwell. After 1 h of incubation at 37 °C in a 5% CO_2_ incubator, the WST-8 sample solution was then transferred to a 96-well plate alongside incubated control wells containing 10 μL CCK-8 solution plus 100 μL of ALI differentiation medium. Absorbance was measured and quantified using a microplate spectrophotometer (Multiskan GO, Thermo Scientific) with SkanIt software, at wavelength 450 nm against background control (blank) and corrected for interference at 650 nm. The CC50 was determined by using GraphPad Prism.

### Time-of-addition assay and withdrawal assay

Time-of-addition assay and drug withdrawal assay were performed following a previously described procedure, with some modifications.^49,50^ For the time-of-addition assay, 200 μM DCM was added at the following time points: (1) within the 8 h period before infection (–8 to –1 h, pretreatment); (2) between 1 h before infection and the time of infection (–1 to 0 h, adsorption); and (3) between 0 and 24 h after infection (replication). Supernatants and cells from the above conditions were respectively harvested 24 h post-infection.

For the drug withdrawal assay, 200 μM DCM was added at the following time points: (1) between 0 and 24 h after infection but removed between 24 and 48 h; (2) between 24 h and 48 h after infection; (3) between 0 and 48 h after infection. Supernatants and cells from the above conditions were respectively harvested at 48 h post-infection.

In addition, DCM is incubated (1) between 0 and 48 h after infection but removed between 48 and 72 h; (2) between 24 h and 72 h after infection; (3) between 48 h and 72 h after infection. Supernatants and cells from the above conditions were respectively harvested at 72 h post-infection.

### SEM and TEM

ALI culture samples with Omicron infection were prepared according to a standard protocol for FEI Quanta 250 FEG-scanning electron microscopy or Hitachi JEM-1400 PLUS-transmission electron microscopy. Representative photomicrographs were taken at various angles to minimise any error in assessment due to specimen tilt or other processing artefacts.

### Bulk RNA sequencing of human AECs

Human primary AECs from three independent donors were treated with ALI medium, 100 μM DCM or 200 μM DCM, which were added to the media on the basolateral side of the culture (Fig. 5a). After 24 h, cells were collected for subsequent high-throughput transcriptome sequencing using an Illumina NovaSeq6000 sequencer. All analyses were performed using R Version 4.0.5 (R Foundation for Statistical Computing, Vienna, Austria).

### Analysis of the single-cell RNA-seq database

The single-cell RNA-seq data for control and COVID-19 patients were obtained from two different databases. The first control database was assembled using the expression matrix and the metadata for the human healthy respiratory tracts downloaded from Deprez et al.^25^ This study involved a database with 77,969 cells from distinct airway locations (the nose, the trachea/carina, intermediate bronchi, and distal bronchi) of 10 healthy volunteers. Values for each sampling location were averaged across donors. Cell types with a total of <250 cells detected were excluded from the analysis.

The second publicly available RNA-seq database of nasopharyngeal swab specimens were taken from patients infected with SARS-CoV-2 through a web portal (https://covid19cellatlas.org), and we generated a database of 13,868 nasal ciliated cells and 13,586 nasal secretory cells from 30 patients in our study. Quality control metrics for our single-cell data are provided at the web portal page. The severity of patients with SARS-CoV-2 infection was divided into four groups (asymptomatic, mild, moderate, and severe) for the analysis of NQO1 expression in ciliated cells and VKORC1 expression in secretory cells.

### Statistical analysis

Statistical analyses were performed using SPSS 21.0 software (IBM, Chicago, IL) and GraphPad Prism. The Kolmogorov-Smirnov tests and Shapiro-Wilk tests revealed that the data were not normally distributed. The Mann-Whitney two-sided nonparametric test was used as appropriate to compare the continuous variables between two groups. *P* < 0.05 was deemed statistically significant for all analyses. All IC50 and CC50 values were generated using GraphPad inhibitor versus response nonlinear regressions.

## Supplementary information

Supplemental figures and tables

Supplementary Data 1

## Data Availability

All research data supporting the findings of this study are available upon reasonable request by readers.

## References

[1] Viana R (2022). Rapid epidemic expansion of the SARS-CoV-2 Omicron variant in southern Africa. Nature.

[2] Du X (2022). Omicron adopts a different strategy from Delta and other variants to adapt to host. Signal Transduct. Target Ther..

[3] Reis S (2022). Nirmatrelvir combined with ritonavir for preventing and treating COVID-19. Cochrane Database Syst. Rev..

[4] Piccicacco N (2022). Real-world effectiveness of early remdesivir and sotrovimab in the highest-risk COVID-19 outpatients during the Omicron surge. J. Antimicrob. Chemother..

[5] Whittington MD, Pearson SD, Rind DM, Campbell JD (2022). The cost-effectiveness of remdesivir for hospitalized patients with COVID-19. Value Health.

[6] De P, Kumar V, Kar S, Roy K, Leszczynski J (2022). Repurposing FDA approved drugs as possible anti-SARS-CoV-2 medications using ligand-based computational approaches: sum of ranking difference-based model selection. Struct. Chem..

[7] Sun C, Zhao W, Wang X, Sun Y, Chen X (2020). A pharmacological review of dicoumarol: An old natural anticoagulant agent. Pharm. Res..

[8] Cheng ST (2021). Dicoumarol, an NQO1 inhibitor, blocks cccDNA transcription by promoting degradation of HBx. J. Hepatol..

[9] Lata S, Ali A, Sood V, Raja R, Banerjea AC (2015). HIV-1 Rev downregulates Tat expression and viral replication via modulation of NAD(P)H:quinine oxidoreductase 1 (NQO1). Nat. Commun..

[10] Timson DJ (2017). Dicoumarol: a drug which hits at least two very different targets in vitamin K metabolism. Curr. Drug Targets.

[11] Aras D, Cinar O, Cakar Z, Ozkavukcu S, Can A (2016). Can dicoumarol be used as a gonad-safe anticancer agent: an in vitro and in vivo experimental study. Mol. Hum. Reprod..

[12] Qiu D (2022). NAD(P)H: quinone oxidoreductase 1 attenuates oxidative stress and apoptosis by regulating Sirt1 in diabetic nephropathy. J. Transl. Med..

[13] Wallin R, Wajih N, Hutson SM (2008). VKORC1: a warfarin-sensitive enzyme in vitamin K metabolism and biosynthesis of vitamin K-dependent blood coagulation factors. Vitam. Horm..

[14] Dofferhoff ASM (2021). Reduced vitamin K status as a potentially modifiable risk factor of severe coronavirus disease 2019. Clin. Infect. Dis..

[15] Luo L (2022). Natural products for infectious microbes and diseases: an overview of sources, compounds, and chemical diversities. Sci. China Life Sci..

[16] Piplani S, Singh P, Winkler DA, Petrovsky N (2022). Potential COVID-19 therapies from computational repurposing of drugs and natural products against the SARS-CoV-2 helicase. Int. J. Mol. Sci..

[17] Zigolo MA, Goytia MR, Poma HR, Rajal VB, Irazusta VP (2021). Virtual screening of plant-derived compounds against SARS-CoV-2 viral proteins using computational tools. Sci. Total Environ..

[18] Whitsett JA (2018). Airway epithelial differentiation and mucociliary clearance. Ann. Am. Thorac. Soc..

[19] Peng Y (2019). Whole-transcriptome sequencing reveals heightened inflammation and defective host defence responses in chronic rhinosinusitis with nasal polyps. Eur. Respir. J..

[20] Modena BD (2017). Gene expression correlated with severe asthma characteristics reveals heterogeneous mechanisms of severe disease. Am. J. Respir. Crit. Care Med..

[21] Lee IT (2020). ACE2 localizes to the respiratory cilia and is not increased by ACE inhibitors or ARBs. Nat. Commun..

[22] Peng Y (2022). Angiotensin-converting enzyme 2 in peripheral lung club cells modulates the susceptibility to SARS-CoV-2 in chronic obstructive pulmonary disease. Am. J. Physiol. Lung Cell Mol. Physiol..

[23] Wu CT (2023). SARS-CoV-2 replication in airway epithelia requires motile cilia and microvillar reprogramming. Cell.

[24] Shytaj IL (2022). The FDA-approved drug cobicistat synergizes with remdesivir to inhibit SARS-CoV-2 replication in vitro and decreases viral titers and disease progression in Syrian hamsters. mBio.

[25] Deprez M (2020). A single-cell atlas of the human healthy airways. Am. J. Respir. Crit. Care Med..

[26] Wu Q (2023). Vaccination effects on post-infection outcomes in the Omicron BA.2 outbreak in Shanghai. Emerg. Microbes Infect..

[27] Wang C (2021). COVID-19 in early 2021: current status and looking forward. Signal Transduct. Target Ther..

[28] Lamontagne F (2020). A living WHO guideline on drugs for covid-19. BMJ.

[29] Pepperrell T, Ellis L, Wang J, Hill A (2022). Barriers to worldwide access for Paxlovid, a new treatment for COVID-19. Open Forum Infect. Dis..

[30] Agarwal S, Agarwal SK (2021). Lopinavir-Ritonavir in SARS-CoV-2 infection and drug-drug interactions with cardioactive medications. Cardiovasc. Drugs Ther..

[31] Yin W (2020). Structural basis for inhibition of the RNA-dependent RNA polymerase from SARS-CoV-2 by remdesivir. Science.

[32] Marrone A (2022). Remdesivir plus dexamethasone versus dexamethasone alone for the treatment of coronavirus disease 2019 (COVID-19) patients requiring supplemental O2 therapy: a prospective controlled nonrandomized study. Clin. Infect. Dis..

[33] Cao Z (2023). VV116 versus nirmatrelvir-ritonavir for oral treatment of Covid-19. N. Engl. J. Med..

[34] Jiang Y (2022). Pharmacological therapies and drug development targeting SARS-CoV-2 infection. Cytokine Growth Factor Rev..

[35] Chocron R (2021). Anticoagulation before hospitalization is a potential protective factor for COVID-19: insight from a French Multicenter Cohort Study. J. Am. Heart Assoc..

[36] Abdel-Qadir H (2022). The association between anticoagulation and adverse outcomes after a positive SARS-CoV-2 test among older outpatients: a population-based cohort study. Thromb. Res.

[37] Hozayen SM (2021). Outpatient and inpatient anticoagulation therapy and the risk for hospital admission and death among COVID-19 patients. EClinicalMedicine.

[38] Rossi R, Coppi F, Talarico M, Boriani G (2020). Protective role of chronic treatment with direct oral anticoagulants in elderly patients affected by interstitial pneumonia in COVID-19 era. Eur. J. Intern Med..

[39] Open SC (2021). Association between warfarin and COVID-19-related outcomes compared with direct oral anticoagulants: population-based cohort study. J. Hematol. Oncol..

[40] Watanabe J (2006). Dicoumarol potentiates cisplatin-induced apoptosis mediated by c-Jun N-terminal kinase in p53 wild-type urogenital cancer cell lines. Oncogene.

[41] Pey AL, Megarity CF, Timson DJ (2019). NAD(P)H quinone oxidoreductase (NQO1): an enzyme which needs just enough mobility, in just the right places. Biosci. Rep..

[42] D’Agnillo F (2021). Lung epithelial and endothelial damage, loss of tissue repair, inhibition of fibrinolysis, and cellular senescence in fatal COVID-19. Sci. Transl. Med..

[43] V’Kovski P, Kratzel A, Steiner S, Stalder H, Thiel V (2021). Coronavirus biology and replication: implications for SARS-CoV-2. Nat. Rev. Microbiol..

[44] Caci E (2009). Epithelial sodium channel inhibition in primary human bronchial epithelia by transfected siRNA. Am. J. Respir. Cell Mol. Biol..

[45] Zhang Y (2007). A transgenic FOXJ1-Cre system for gene inactivation in ciliated epithelial cells. Am. J. Respir. Cell Mol. Biol..

[46] Goswami R (2021). Oral Hsp90 inhibitor SNX-5422 attenuates SARS-CoV-2 replication and dampens inflammation in airway cells. iScience.

[47] Plaze M (2021). Inhibition of the replication of SARS-CoV-2 in human cells by the FDA-approved drug chlorpromazine. Int J. Antimicrob. Agents.

[48] Yu T (2021). Assessment of benchmark dose in BEAS-2B cells by evaluating the cell relative viability with particulates in motorcycle exhaust via the air-liquid interface exposure. Biomed. Environ. Sci..

[49] Varghese FS (2021). Berberine and Obatoclax inhibit SARS-Cov-2 replication in primary human nasal epithelial cells in vitro. Viruses.

[50] Senaweera S (2022). Discovery of N-benzyl hydroxypyridone carboxamides as a novel and potent antiviral chemotype against human cytomegalovirus (HCMV). Acta Pharm. Sin. B.

