# Supplementary material for: Dicoumarol is an effective post-exposure prophylactic for SARS-CoV-2 Omicron infection in human airway epithelium
Source: Signal Transduct Target Ther. 2023 Jun 10;8:242. doi: 10.1038/s41392-023-01511-7 (PMC10256976; doi:10.1038/s41392-023-01511-7)
Supplement: Supplementary file 1 — Supplemental figures and tables [file 41392_2023_1511_MOESM1_ESM.docx]

Supplementary Materials for

Dicoumarol is an effective post-exposure prophylactic for SARS-CoV-2 Omicron infection in human airway epithelium

Yang Peng, Shi-ying Chen, Zhao-ni Wang, Zi-qing Zhou, Jing Sun, Gui-an Zhang, Jia Li, Lei Wang, Jin-cun Zhao, Xiao Xiao Tang, De-Yun Wang, Nan-shan Zhong

**Correspondence to**: Nan-shan Zhong (nanshan@vip.163.com); De-Yun Wang (entwdy@nus.edu.sg); Xiao Xiao Tang (tangxiaoxiao@gird.cn).

**This PDF file includes:**

Figures. S1 to S4

Tables S1 to S2

Data S1


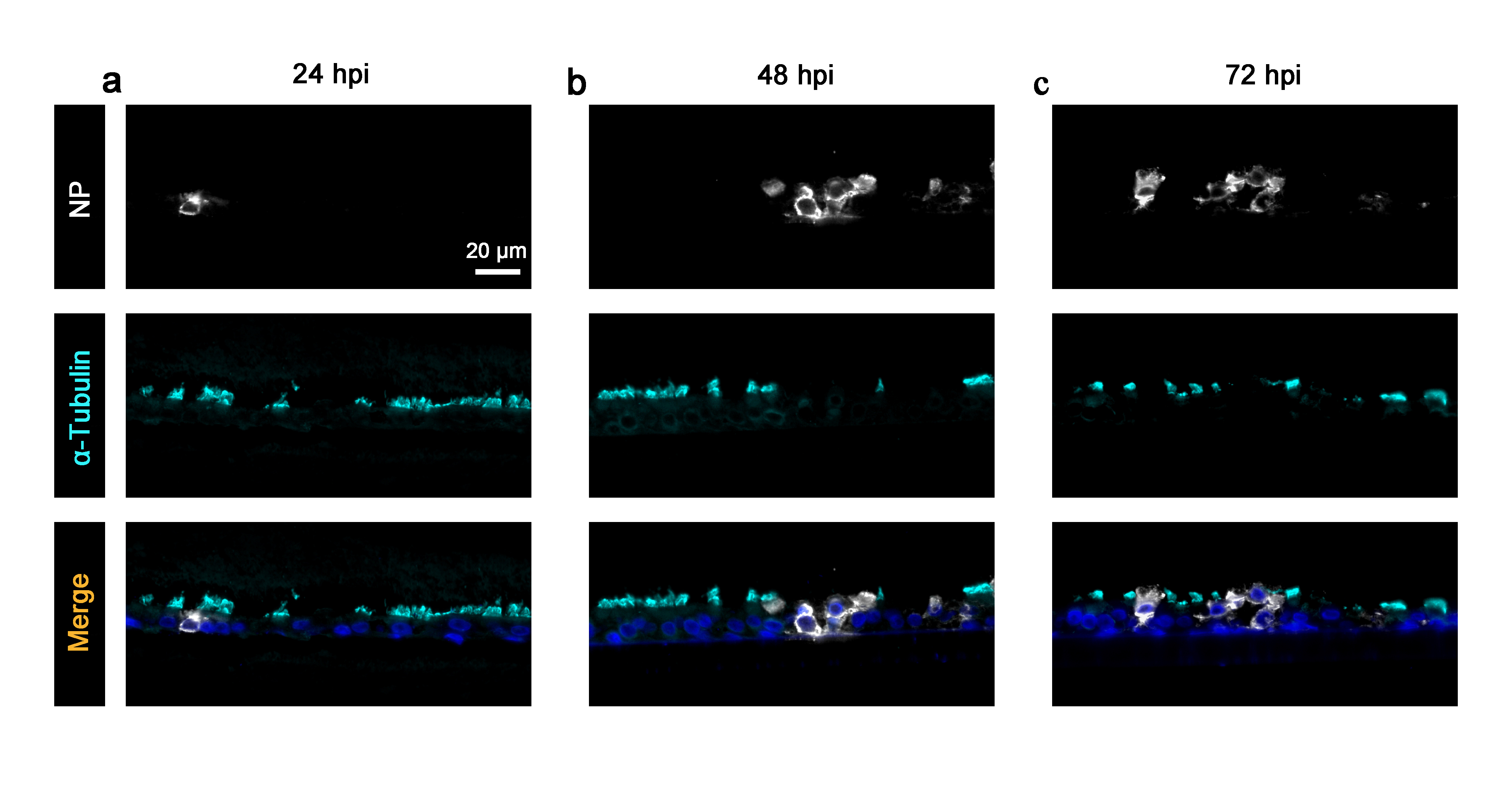


**Figure S1. The characteristics of Omicron infection in primary human small airway epithelial cells**

Representative immunofluorescence images showed the characteristics of primary human small airway epithelial cells infected with Omicron BA.1 at a multiplicity of infection of 0.1 at 24 (**a**), 48 (**b**) and 72 (**c**) hpi, respectively.

Abbreviations: hpi, hours post infection.


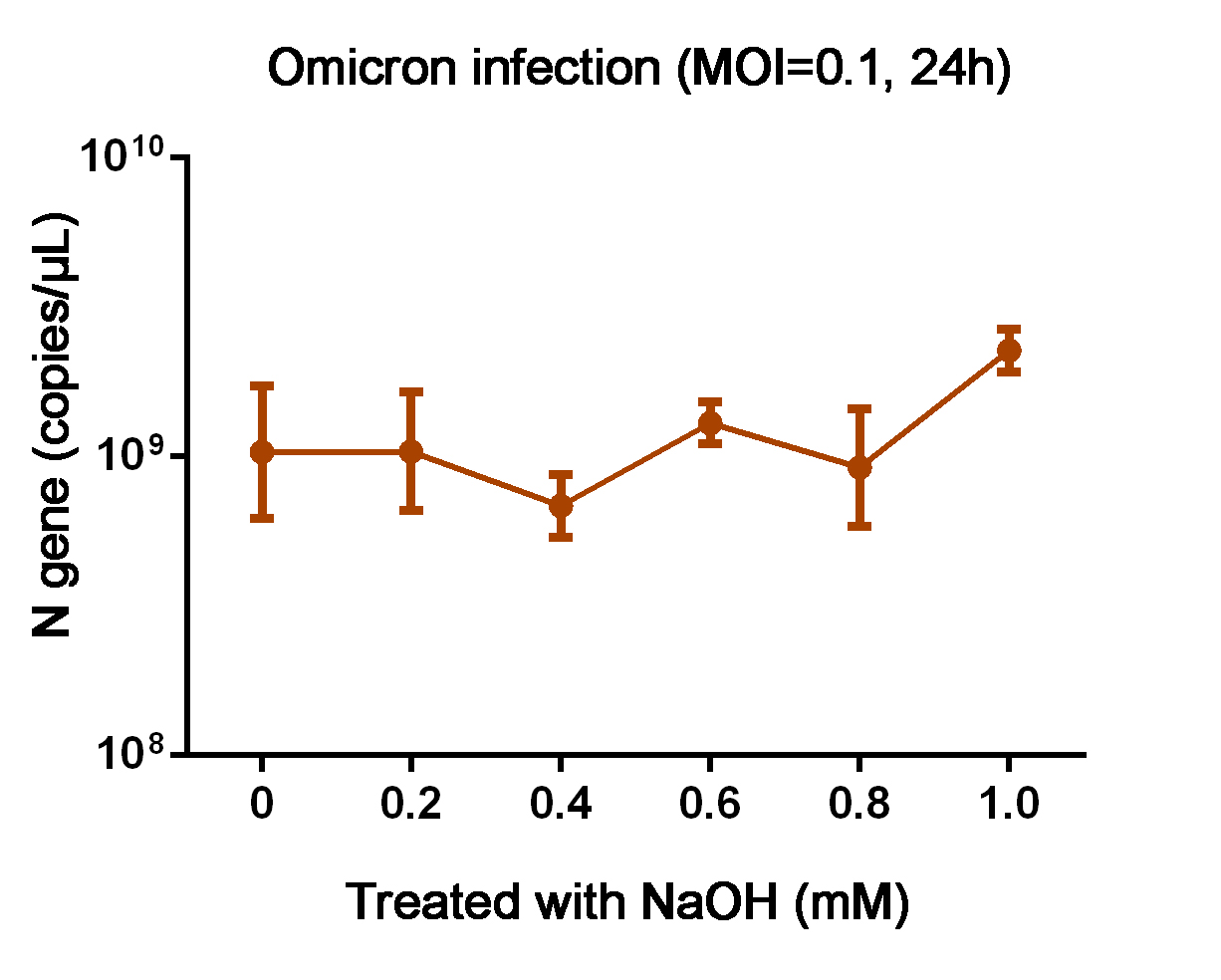


**Figure S2. NaOH treatment did not inhibit Omicron infection in AECs**

NaOH at concentration of 0, 0.2, 0.4, 0.6, 0.8 and 1.0 mM (solution for dicoumarol at 0, 50, 100, 150, 200 and 250 μM) did not inhibit the replication of Omicron in cultured primary AECs at 24 h post infection.

Abbreviations: AECs, airway epithelial cells; MOI, multiplicity of infection.


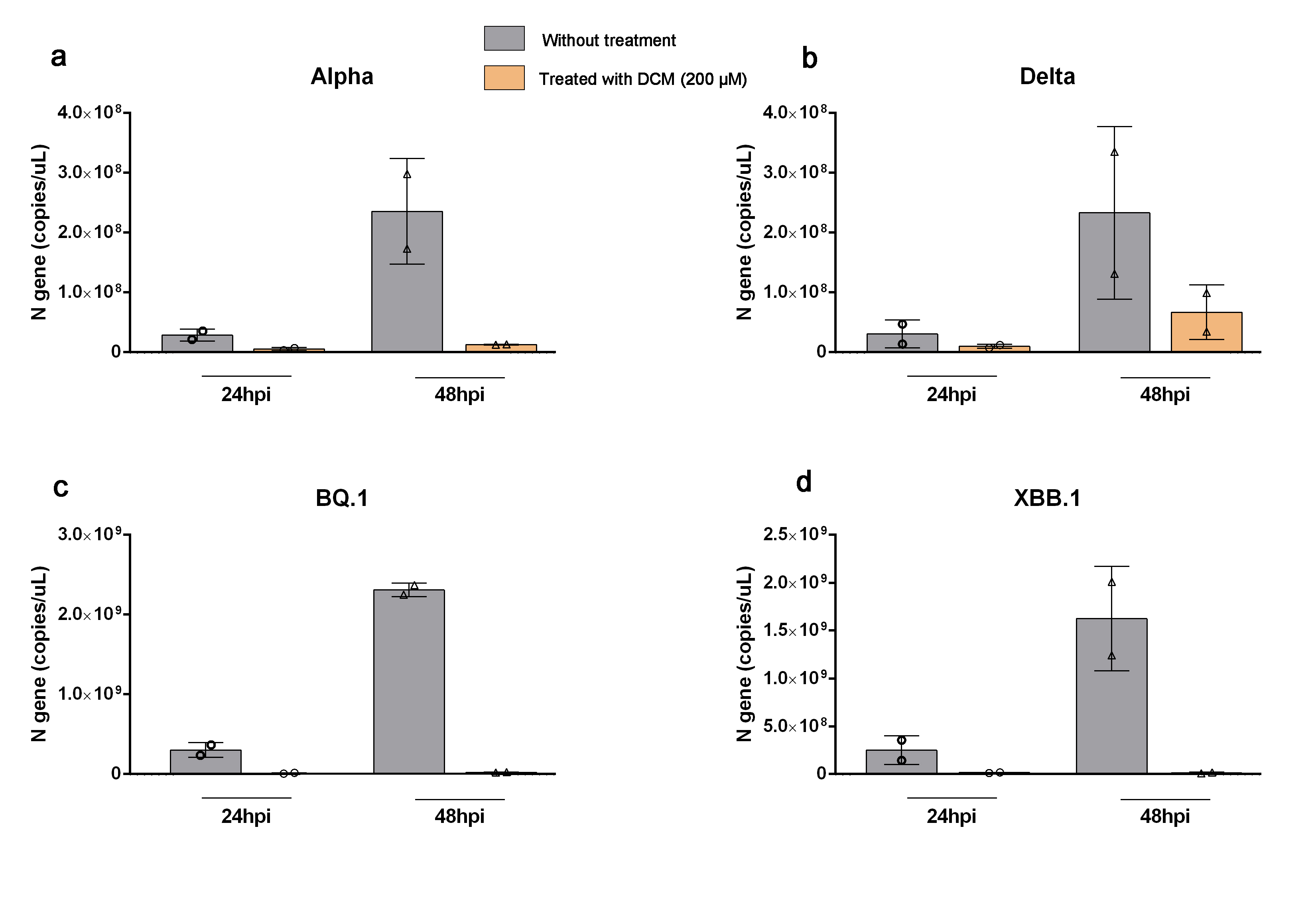
**Figure S3.** **DCM treatment inhibited multiple SARS-CoV-2 variants in AECs**

DCM treatment at a concentration of 200 μM could inhibit the infection of multiple SARS-CoV-2 variants [including Alpha (**a**), Delta (**b**) Omicron BQ.1 (**c**) and Omicron XBB.1 (**d**)] in cultured AECs (n=2) at 24 hpi or 48 hpi.

Abbreviations: AECs, airway epithelial cells; DCM, dicoumarol; hpi, hours post infection; MOI, multiplicity of infection.


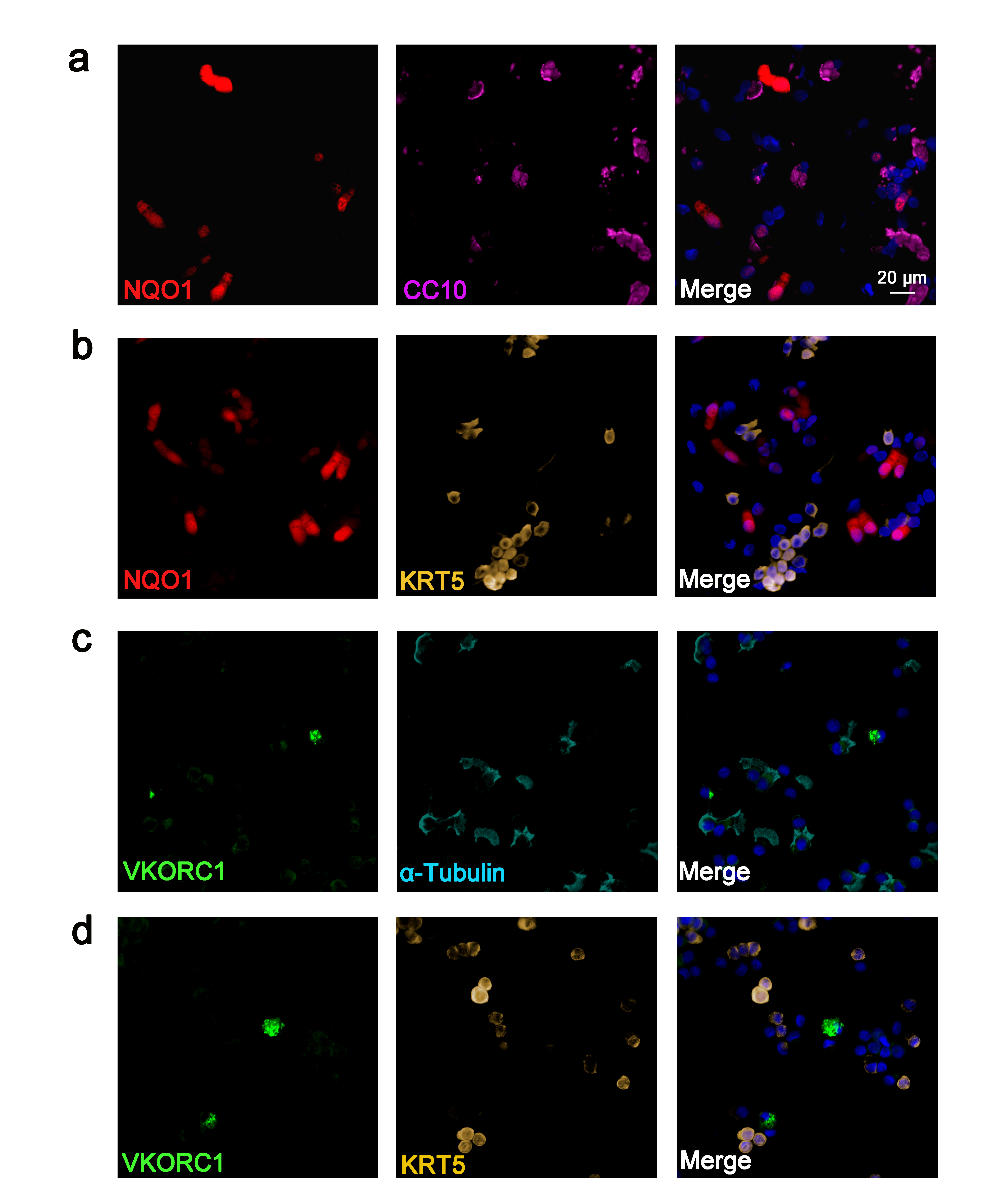


**Figure S4. The localization of NQO1 and VKORC1 protein in primary AECs**

Representative immunofluorescence images in cytospin experiment revealed that NQO1 was not expressed in CC10^+^ secretory cells (**a**) or KRT5^+^ basal cells (**b**), and VKORC1 was not expressed in α-tubulin^+^ ciliated cells (**c**) or KRT5^+^ basal cells (**d**).

Abbreviations: AECs, airway epithelial cells; CC10, club cell 10 kDa protein; KRT5, Keratin 5; NQO1, NAD(P)H quinone oxidoreductase 1; VKORC1, Vitamin K epoxide reductase complex subunit 1.

**Table S1. The pH value of culture medium treated with DCM or NaOH**

| Concentration of DCM (μM) | 0 | 50 | 100 | 200 | 400 |
| --- | --- | --- | --- | --- | --- |
| Mean pH value | 8.18 | 8.02 | 8.22 | 8.22 | 8.06 |
|  |  |  |  |  |  |
| Concentration of NaOH (mM) | 0 | 0.5 | 1.0 | 2.0 | 4 |
| Mean pH value | 8.18 | 8.31 | 8.39 | 8.30 | 8.39 |

Abbreviations: DCM, dicoumarol.

**Table S2. List of antibodies**

| Reagent | Supplier | Identifier (Cat#) | Clonality (Isotype) | Diluted concentration |
| --- | --- | --- | --- | --- |
| α-Tubulin | Abcam | ab89984 | Polyclonal (IgY) | 1:150 |
| α-Tubulin | Abcam | ab24610 | Monoclonal (IgG2b) | 1:600 |
| CC10 | Santa | sc-365992 | Monoclonal (IgG1) | 1:500 |
| Keratin 5 | Biolegend | 905903 | Polyclonal (IgY) | 1:150 |
| NQO1 | Abcam | ab80588 | Monoclonal (IgG) | 1:100 |
| SARS-CoV Nucleoprotein | Sino Biological | 40143-MM08 | Monoclonal (IgG1) | 1:1000 |
| SARS-CoV Nucleoprotein | Rockland | 600-401-A50 | Polyclonal (IgG) | 1:2000 |
| VKORC1 | Abcam | ab206656 | Monoclonal (IgG) | 1:100 |

Abbreviations: CC10, club cell 10 kDa protein; NQO1, NAD(P)H quinone oxidoreductase 1; VKORC1, Vitamin K epoxide reductase complex subunit 1.

**Data S1. (Separate file)**

A list highlights markers distinguishing broad cell categories based on average expression level in the human airway. [from a published resource (PMID: 32726565) SUPPLEMENTARY TABLE E6a]
